# Gastric perforation at the extremes of age: a comparative case report of NSAID-associated high-grade dysplasia versus methamphetamine-induced ischemic perforation

**DOI:** 10.1186/s12245-026-01218-w

**Published:** 2026-04-14

**Authors:** Aisel Paul, Mohamed Hamza, N. Kiran, L. T. Sunandhakumari

**Affiliations:** ¹Department of General Surgery, Sree Gokulam Medical College & Research Foundation, Venjaramoodu P.O, Thiruvananthapuram, Kerala PIN-695607 India

**Keywords:** Peptic ulcer perforation, Anti-inflammatory agents, Non-steroidal, Methamphetamine, Ischemia, Substance-related disorders, Precancerous conditions

## Abstract

**Background:**

Gastric perforation is a surgical emergency with evolving etiologies. While traditionally linked to the use of Non-Steroidal Anti-Inflammatory Drugs (NSAIDs) in elderly individuals, a rising incidence in young adults is increasingly associated with polysubstance use. This report compares two distinct cases to highlight the demographic and pathophysiological dichotomy facing surgeons today.

**Case report:**

We present two contrasting cases of gastric perforation. The first is an 85-year-old male with chronic NSAID use who presented with an acute perforation, with histopathology of the ulcer edge unexpectedly revealing high-grade dysplasia. The second case involves a 32-year-old male with a history of polysubstance use, including methamphetamine, who presented with a diagnostically challenging ischemic perforation that required computed tomography for diagnosis following an initially negative upright abdominal radiograph. Both patients were successfully managed with modified Graham patch omentoplasty.

**Discussion:**

These cases represent fundamentally different disease processes: one driven by chronic prostaglandin inhibition leading to a premalignant lesion, and the other by acute sympathomimetic-induced vasoconstriction resulting in ischemic necrosis. This comparison underscores two critical lessons: the importance of maintaining a high index of suspicion and the liberal use of cross-sectional imaging in young patients with polysubstance use, and the non-negotiable mandate for routine ulcer edge biopsy in all perforation cases to identify underlying pathology such as dysplasia. Management must extend beyond surgical repair to include oncological surveillance or addiction rehabilitation, tailored to the underlying etiology.

## Background

Perforated peptic ulcer (PPU) is a life-threatening surgical emergency associated with significant morbidity and mortality, with reported short-term mortality rates reaching approximately 30% and morbidity affecting up to 50% to 60% of patients [1]. While the overall incidence of uncomplicated peptic ulcer disease has declined in recent decades, the burden of complicated ulcers requiring emergency intervention remains substantial, with annual incidence estimates for perforation ranging from 3.8 to 14 per 100,000 individuals [2].

Traditionally, Helicobacter pylori infection and the use of non-steroidal anti-inflammatory drugs (NSAIDs) have been the predominant etiologies, primarily affecting older populations. A systematic review by Amalia et al. (2024) reported that the overall prevalence of H. pylori infection among patients with gastroduodenal perforation was 58%, underscoring its continued significance [3]. NSAID use represents another critical risk factor, with studies indicating that approximately 50% of gastroduodenal ulcer complications in certain populations are NSAID-related [3]. The typical demographic for these etiologies is the elderly population, generally defined as individuals aged 65 years and older, who often have multiple comorbidities and are exposed to NSAIDs for chronic musculoskeletal conditions [4].

However, the epidemiology of PPU is shifting, with recent data revealing a rising incidence among young adults under 40 years of age, particularly men [5, 6]. This change is often linked to lifestyle factors, including alcohol and illicit drug use. Alcohol consumption is a recognized risk factor that can directly erode the gastric mucosal barrier; a population-based cohort study demonstrated that alcohol intake significantly increases the risk of perforated peptic ulcer [7].

Furthermore, methamphetamine use is an emerging and critical cause of gastrointestinal catastrophe through severe ischemic mechanisms. Methamphetamine is a potent sympathomimetic that induces profound splanchnic vasoconstriction, leading to focal ischemia and, in severe cases, perforation [8]. In a case series by Anderson et al. (2018), ten patients with methamphetamine use presented with severe non-occlusive mesenteric ischemia; among these, three had perforated duodenal ulcers, and the overall mortality rate was 60% [9]. This emerging trend is of particular concern in emergency medicine, where a high index of suspicion is required for diagnosis [8, 9].

This report contrasts two distinct cases of gastric perforation—one classic NSAID-induced perforation in an elderly patient (aged ≥ 65 years) and one methamphetamine-associated ischemic perforation in a young adult (aged < 40 years)—to highlight the divergence in pathophysiology, diagnostic challenges, and management strategies required for these populations. By comparing these extremes of age and etiology, we aim to provide practical clinical insights for emergency physicians and surgeons facing this evolving disease pattern.

## Case report

### Patient 1: NSAID-Induced Perforation with High-Grade Dysplasia

An 85-year-old man with a history of chronic diclofenac use for osteoarthritis presented with a three-day history of severe, generalized epigastric pain. On examination, he had fever (38.5 °C), tachycardia (110 bpm), and hypotension (90/60 mmHg). The abdomen was diffusely tender with board-like rigidity, consistent with generalized peritonitis.

**Fig. 1:**
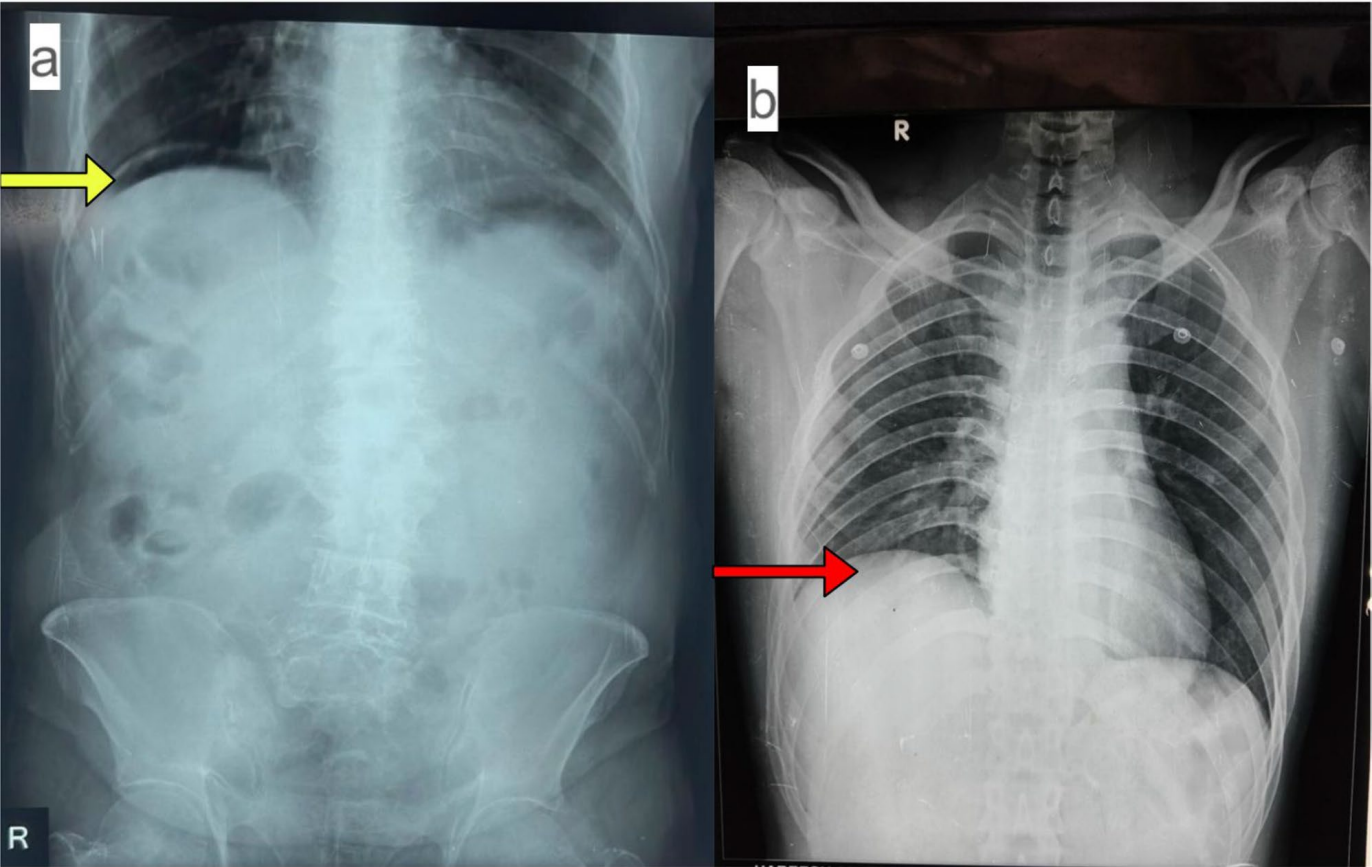
Upright abdominal radiographs. (**a**) Patient 1: Presence of subdiaphragmatic free air (yellow arrow). (**b**) Patient 2: Absence of visible subdiaphragmatic free air (red arrow)

Laboratory studies revealed a white blood cell count of 18,500 cells/mm³. An upright abdominal radiograph showed significant subdiaphragmatic pneumoperitoneum (Fig. 1a). In the emergency department, resuscitation was initiated with 1.5 L of balanced crystalloid solution and intravenous piperacillin-tazobactam. The patient was stabilized without vasopressors or endotracheal intubation; no cardiopulmonary resuscitation was required. He was promptly taken to the operating room for an emergency exploratory laparotomy.

**Fig. 2:**
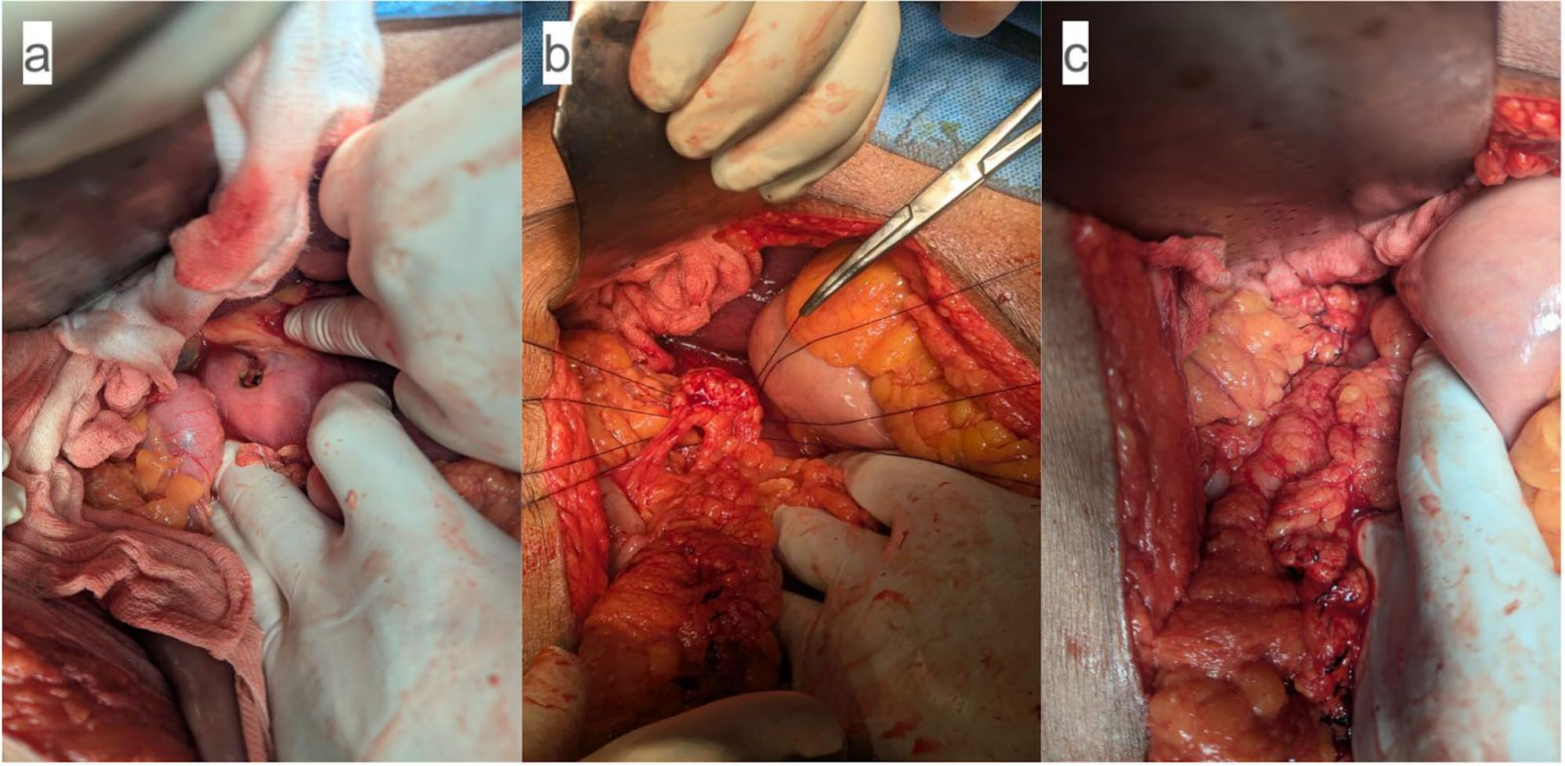
Intraoperative omentoplasty in Patient 1. (**a**) 1 × 1 cm perforation in the prepyloric antrum. (**b**) Placement of a pedicled omental patch over the defect. (**c**) Completed repair secured with interrupted silk sutures

Intraoperatively, a 1 × 1 cm perforation was found on the anterior prepyloric antrum with generalized contamination and approximately 500 mL of purulent fluid. The ulcer edges were thickened and indurated, suggesting a chronic process. After thorough peritoneal lavage with 3 L of warmed normal saline, an ulcer edge biopsy was obtained. Using standard laparotomy instruments, the perforation was repaired with a modified Graham patch omentoplasty using interrupted 2‑0 silk sutures to secure a pedicled omental patch (Fig. 2). A 28-Fr closed suction drain was placed in the subhepatic space. Because of the patient’s advanced age and anticipated catabolic stress from sepsis, a feeding jejunostomy was placed for early enteral nutrition.

The postoperative course was uneventful. He spent three days in the intensive care unit (ICU) and seven days in the ward before discharge. He was discharged on an empirical *Helicobacter pylori* eradication regimen (amoxicillin, clarithromycin, and a proton pump inhibitor) as per institutional protocol.

**Fig. 3:**
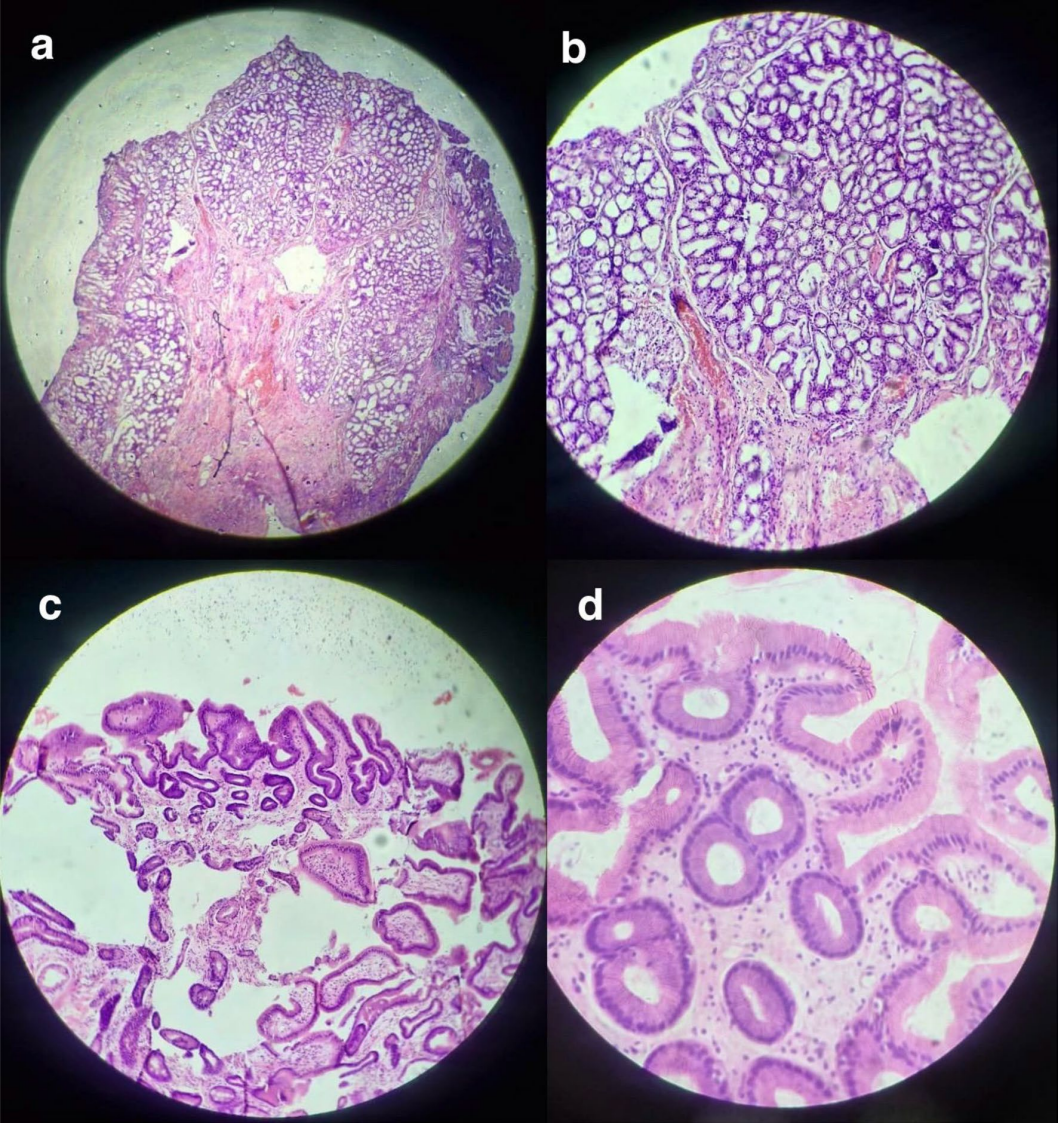
Histopathological findings in Patient 1 (hematoxylin and eosin stain). (**a**) Low-power view showing ulcerated gastric mucosa with necrosis, hemorrhage, and underlying granulation tissue. (**b**) Higher magnification demonstrating distorted glandular architecture with nuclear atypia, consistent with high-grade dysplasia. (**c**) Low-power view of follow-up endoscopic biopsy showing gastric mucosa with areas of intestinal metaplasia. (**d**) Higher magnification demonstrating intestinal metaplasia with goblet cells and foveolar epithelium; no dysplasia or malignancy is identified

Histopathological examination of the intraoperative biopsy revealed ulcerated gastric mucosa with extensive necrosis and hemorrhage at the perforation site. The ulcer base showed fibrino-necrotic debris, granulation tissue, and distorted glandular architecture. Focally, irregular, crowded dysplastic glands with nuclear atypia and loss of polarity within the lamina propria were identified, consistent with true high-grade dysplasia (Fig. 3a, b). Focal features suggestive of chemical gastropathy (foveolar hyperplasia and lamina propria edema) were also noted. No invasive carcinoma was identified.

**Fig. 4:**
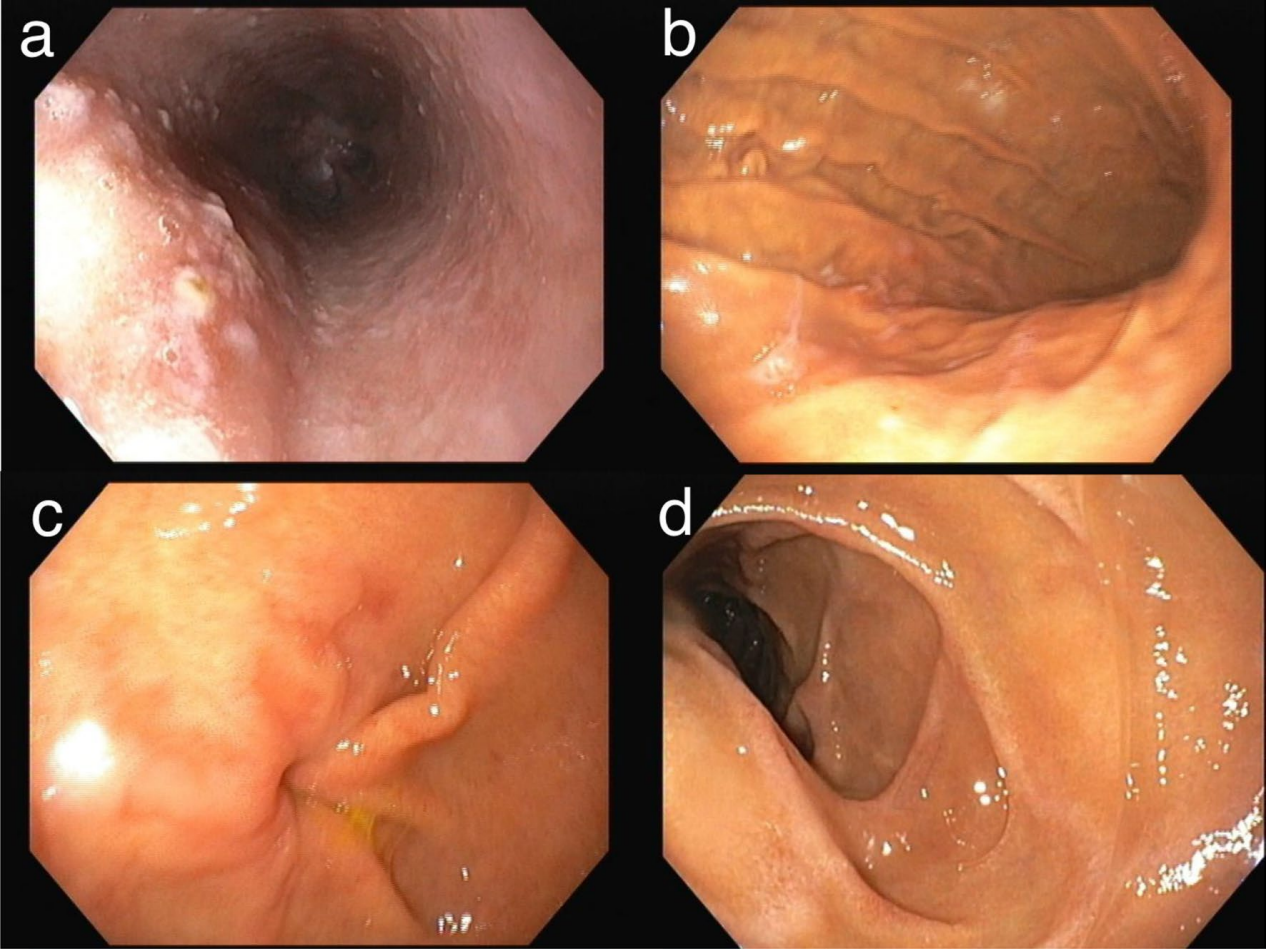
Esophagogastroduodenoscopy (EGD) findings in Patient 1. (**a**) Distal esophagus showing linear mucosal erosions consistent with erosive esophagitis. (**b**) Gastric body showing mild mucosal erythema. (**c**) Pyloric antrum showing patchy erythema and mucosal irregularity corresponding to the biopsy site. (**d**) Duodenum (D1–D2 junction) showing luminal narrowing without evidence of active ulceration

A follow-up esophagogastroduodenoscopy (EGD) performed 10 weeks later showed small esophageal varices, Grade B erosive esophagitis, and patchy erythema with mucosal nodularity in the distal antrum and pyloric region (Fig. 4). Biopsies from the antrum and lesser curvature demonstrated intestinal metaplasia with goblet cells and foveolar epithelium, along with mild inflammatory infiltrate (Fig. 3c, d). No residual dysplasia or malignancy was found.

The patient was advised to permanently discontinue diclofenac and to continue proton pump inhibitor therapy (pantoprazole 40 mg once daily) for gastroprotection. He was referred to the gastroenterology department for ongoing clinical follow-up.

Given his advanced age (85 years), the absence of residual dysplasia on repeat biopsies, and the potential risks associated with repeated endoscopic procedures, a conservative surveillance strategy was adopted. Routine surveillance EGD was not scheduled; instead, repeat endoscopy will be performed only if clinically indicated, such as the development of new or progressive symptoms (e.g., dysphagia, unexplained weight loss, or gastrointestinal bleeding), or at the discretion of the treating gastroenterologist based on changes in clinical status. He remains under periodic outpatient follow-up with symptom-directed evaluation.

### Patient 2: Methamphetamine-Induced Ischemic Perforation

A 32-year-old male presented with a two-week history of abdominal pain that acutely worsened over the preceding three days, progressing from a dull ache to severe, generalized pain. He also reported coffee-ground vomiting. He had used methamphetamine two weeks prior, followed by three days of anorexia and the insidious onset of pain. His social history included smoking for eight years and frequent use of alcohol and cannabis for over a decade.

He initially presented to an outside facility, where an upright abdominal radiograph did not reveal free intraperitoneal air (Fig. 1b). He was diagnosed with acute gastritis and managed with a single intravenous dose of pantoprazole 40 mg, intravenous paracetamol 1 g, and dextrose normal saline infusion, following which he was discharged. This led to a diagnostic delay of approximately 24 h before he re-presented to our center with worsening abdominal pain.

**Fig. 5:**
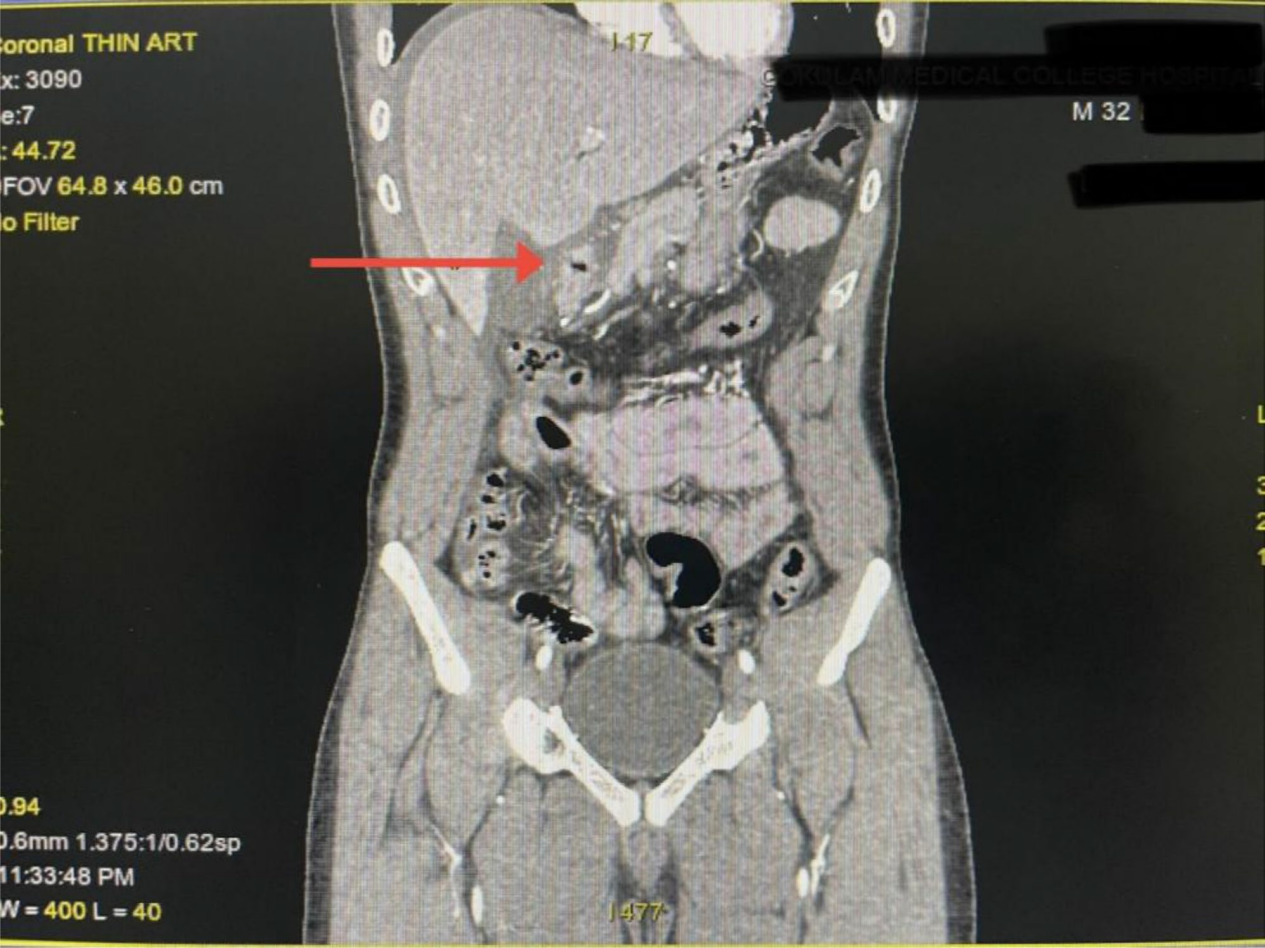
Coronal CT scan of Patient 2 demonstrating a gastric perforation (red arrow) with localized pneumoperitoneum

Upon arrival at our center, he was hemodynamically stable, but abdominal examination revealed board-like rigidity and absent bowel sounds. His white blood cell count was 12,000 cells/mm³. An ultrasound showed moderate free fluid. Given the clinical mismatch—severe peritoneal signs despite a negative initial radiograph—a contrast-enhanced computed tomography (CT) scan was performed. The CT demonstrated a focal gastric wall defect near the pylorus with localized contamination and minimal pneumoperitoneum, consistent with a perforation (Fig. 5).

**Fig. 6:**
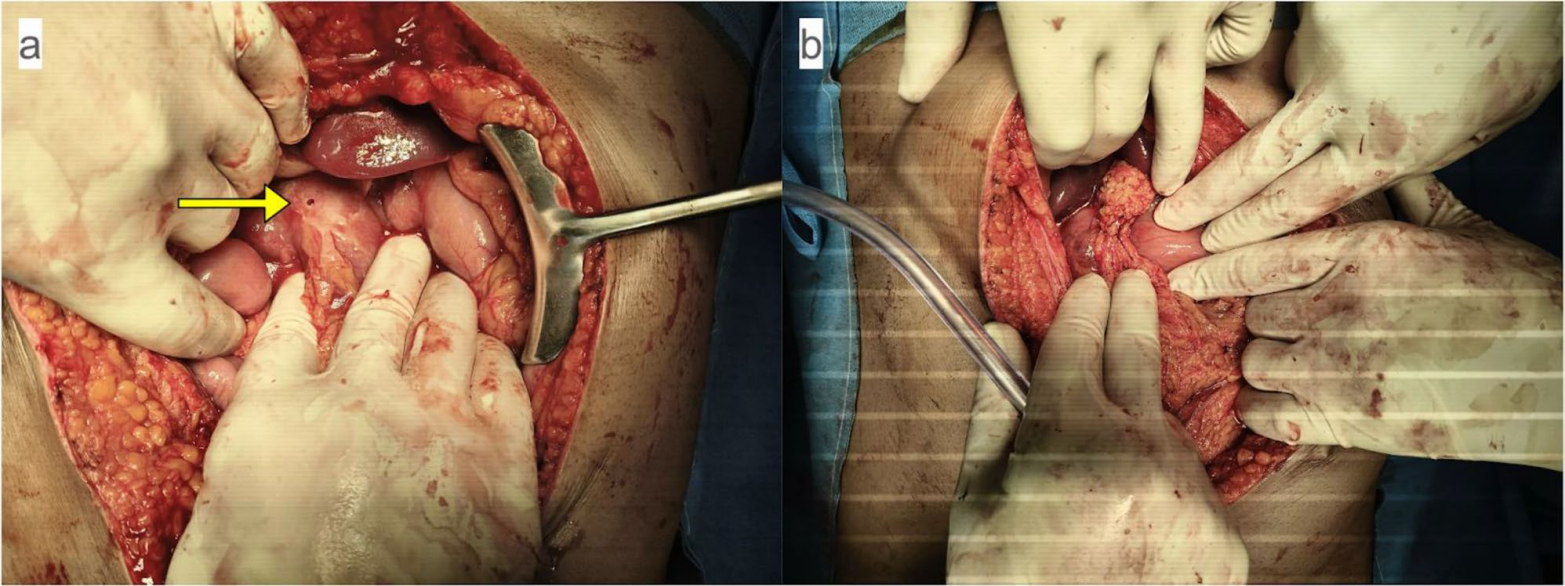
Intraoperative findings in Patient 2. (**a**) 0.5 × 0.5 cm perforation on the anterior pyloric wall (yellow arrow) with pale, ischemic margins. (**b**) Completed modified Graham patch repair

The patient underwent an emergency exploratory laparotomy. Intraoperatively, a well-circumscribed round perforation measuring 0.5 × 0.5 cm was identified on the anterior pyloric wall, with dusky, edematous margins suggestive of ischemic insult (Fig. 6a). Localized contamination with minimal free fluid was present. A thorough peritoneal lavage with 3 L of warmed normal saline was performed, and an edge biopsy from the perforation site was obtained. Using standard laparotomy instruments, the perforation was repaired with a modified Graham patch omentoplasty using interrupted 2‑0 silk sutures to secure a pedicled omental patch (Fig. 6b). A 28-Fr closed suction drain was placed adjacent to the repair. A feeding jejunostomy was not placed because of the patient’s young age and the absence of pre-existing malnutrition (body mass index 23 kg/m², serum albumin 3.8 g/dL) or prolonged catabolic stress.

Given his history of polysubstance use, potential complications such as withdrawal symptoms (e.g., agitation, autonomic instability), surgical site infection, repair site leak, and intra-abdominal abscess formation were anticipated. However, the patient remained clinically stable throughout hospitalization and did not exhibit withdrawal symptoms. The postoperative course was uneventful, and he was discharged on postoperative day 7 with an empirical Helicobacter pylori eradication regimen (amoxicillin, clarithromycin, and a proton pump inhibitor) as per institutional protocol.

**Fig. 7:**
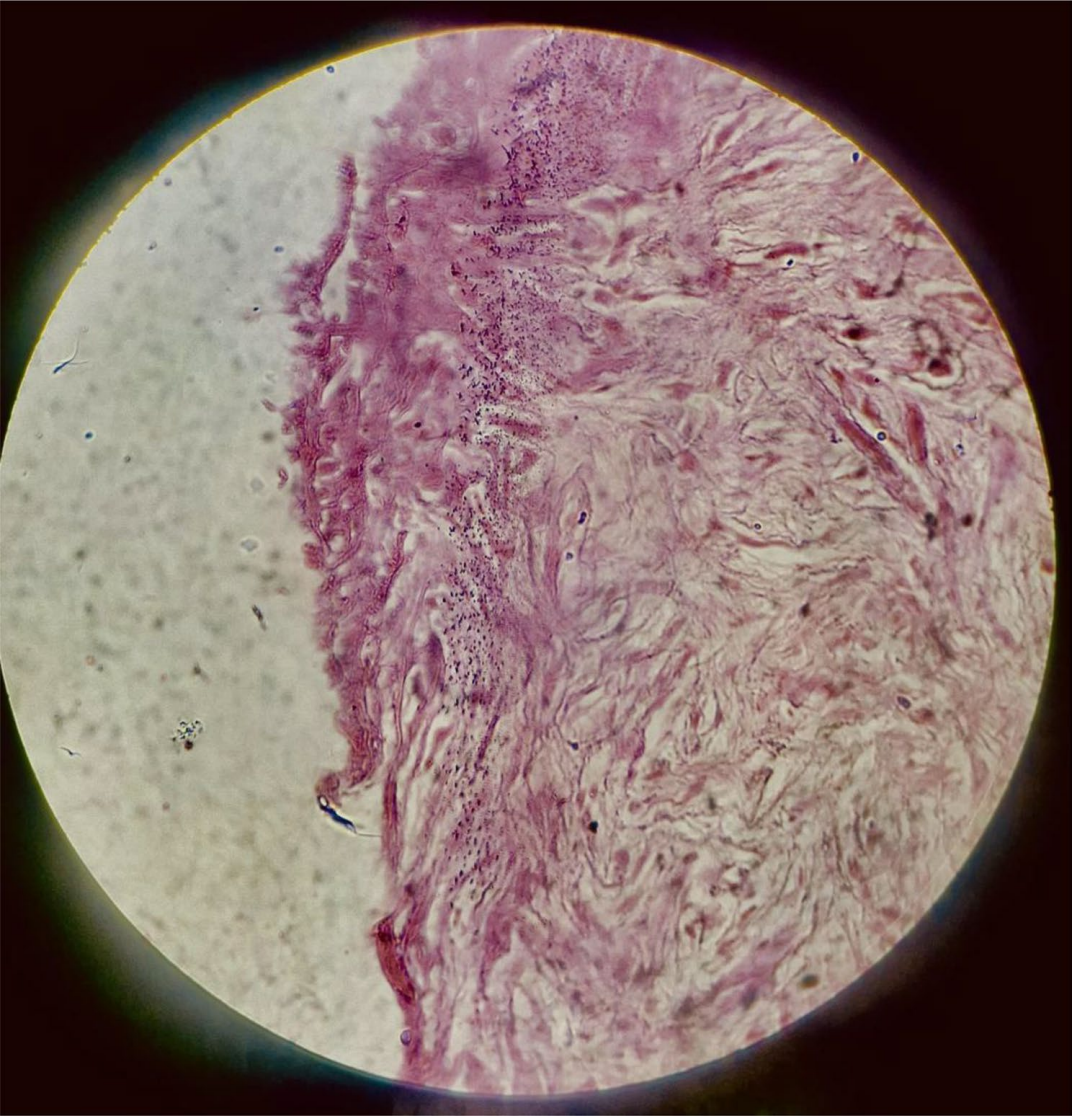
Histopathological findings in Patient 2 (hematoxylin and eosin stain). Sections show fragments of fibrocollagenous tissue with coagulative necrosis and dense neutrophilic infiltration. No intact gastric mucosa is identified, consistent with acute ischemic injury. No dysplasia or malignancy is seen

Histopathological examination of the intraoperative biopsy revealed fragments of fibrocollagenous tissue with myxoid change, dense neutrophilic infiltration, and areas of coagulative necrosis; no intact mucosal lining was identified (Fig. 7). These findings were consistent with acute inflammation and ischemic injury. No dysplastic or malignant changes were present.

The patient was referred to addiction rehabilitation services upon discharge but was subsequently lost to follow-up despite referral.

## Discussion

These two cases, while both culminating in gastric perforation, represent fundamentally different disease processes and highlight the importance of considering patient demographics and risk factors in diagnosis and management, as summarized in Table 1.

**Table 1:** Comparative analysis of clinical and pathological features

Feature	Case 1	Case 2
Demographics	85-year-old male	32-year-old male
Primary Etiology	Chronic NSAID Use (diclofenac)	Polysubstance Abuse (methamphetamine, cannabis, alcohol)
Symptom Duration	Acute onset, 3 days	Insidious onset, 2 weeks
Clinical Signs	Peritonitis with systemic toxicity (Hypotension)	Peritonitis with hemodynamic stability
Initial Imaging	Radiograph: Pneumoperitoneum positive	Radiograph: Pneumoperitoneum negative
Confirmatory Imaging	Not required	Contrast-Enhanced CT Scan
Perforation Site	Prepyloric antrum, 1 × 1 cm	Anterior Pyloric Wall, 0.5 × 0.5 cm
Intraoperative appearance	Indurated edges with generalized contamination	Well-circumscribed round perforation with localized contamination
Surgical Repair	Modified Graham patch Omentoplasty	Modified Graham patch Omentoplasty
Additional procedures	Feeding Jejunostomy	None
Histopathological findings	Chronic inflammation with High-Grade Dysplasia	Acute ischemic necrosis; no dysplasia
Postoperative Course	Uneventful (10 days total)	Uneventful (7 days total)
Pathophysiology	Chronic NSAID-induced mucosal injury with dysplastic transformation	Acute ischemic injury secondary to methamphetamine-induced vasoconstriction
Long-term management plan	Oncological surveillance (Endoscopy)	Addiction rehabilitation and counseling

### Diagnostic challenges and radiological discrepancies

A key lesson from this comparison is the variability in clinical and radiological presentation. The elderly patient (Case 1) presented with classic, hyperacute peritonitis and was readily diagnosed using plain radiography, which demonstrated clear pneumoperitoneum. In contrast, the young patient (Case 2) had an insidious onset and a negative initial radiograph.

Plain radiographs fail to detect pneumoperitoneum in up to 30% of perforation cases [10]. In young patients with polysubstance use, this limitation may be further accentuated, as ischemic perforations are often smaller and may be rapidly contained by the omentum, limiting the volume of free air. In Case 2, the critical diagnostic clue was the discordance between a negative radiograph and overt peritoneal signs. This underscores an important clinical principle: a negative radiograph does not exclude perforation in high-risk patients. A low threshold for cross-sectional imaging is essential, and computed tomography (CT) remains the gold standard for identifying contained perforations and guiding timely surgical intervention [10].

### Pathophysiological differences: inflammation versus ischemia

The histopathological findings in these cases reflect two distinct mechanisms of injury. In the elderly patient, the perforation represents the culmination of a chronic inflammatory process driven by NSAID use. NSAIDs induce mucosal injury through both systemic inhibition of gastroprotective prostaglandins via cyclooxygenase‑1 (COX‑1) inhibition and direct topical mucosal damage [4, 11, 12], creating an environment prone to dysplasia.

In contrast, the perforation in the young patient was driven by acute vascular compromise. Methamphetamine, a potent sympathomimetic, induces profound splanchnic vasoconstriction via catecholamine excess, resulting in focal ischemia [8, 9, 13]. The intraoperative finding of a well‑circumscribed round perforation with dusky margins, along with histological evidence of coagulative necrosis and absence of normal mucosa, supports an ischemic etiology. Unlike chronic peptic ulcers—which demonstrate a zoned architecture with granulation tissue and fibrosis—methamphetamine‑related lesions show abrupt necrosis with minimal chronic inflammatory scarring [8, 14, 15]. This ischemic insult may be further exacerbated by concurrent alcohol use, which impairs mucosal defense mechanisms [7].

### Implications for management and surveillance

Management of gastric perforation extends beyond surgical repair and must be tailored to the underlying etiology.

From a surgical perspective, in both patients, the perforation was successfully managed with a modified Graham patch omentoplasty following thorough peritoneal lavage. Adjunctive procedures were individualized: a feeding jejunostomy was performed in the elderly patient to support early enteral nutrition, whereas it was avoided in the younger patient due to adequate nutritional status and physiological reserve.

#### Case 1

Oncological surveillance: The incidental finding of high‑grade dysplasia (HGD) transformed this case into an oncological concern. Although malignancy is uncommon, up to 13% of perforated peptic ulcers may harbor gastric cancer, supporting the routine use of ulcer edge biopsy [1]. Current guidelines differ in recommended surveillance strategies: the European Society of Gastrointestinal Endoscopy (ESGE) MAPS II guidelines recommend that patients with HGD in the absence of an endoscopically defined lesion undergo immediate high‑quality endoscopic reassessment; if no lesion is found, surveillance endoscopy is advised at 6 months [16]. For low‑grade dysplasia (LGD), the MAPS II guidelines suggest surveillance at 12 months after an initial negative high‑quality examination. In contrast, some Asian perspectives, including a 2016 Korean review, emphasize a more aggressive interventional approach, recommending endoscopic resection for HGD given the high risk of progression to or coexistence with invasive carcinoma [17]. In this patient, advanced age (85 years) and absence of residual dysplasia on follow‑up biopsies justified a conservative, symptom‑directed surveillance strategy, balancing oncological risk against procedural burden.

Long‑term proton pump inhibitor therapy in elderly patients carries recognized risks, including reduced bone mineral density, enteric infections (e.g., *Clostridioides difficile*), and micronutrient deficiencies [18–20]. However, in this case, the benefits of secondary prophylaxis and mucosal healing outweighed these risks, particularly given the history of perforation and ongoing requirement for gastroprotection.

#### Case 2

Addiction rehabilitation: In the younger patient, the principal long‑term risk was ongoing polysubstance use. Management therefore extends beyond surgery and requires a multidisciplinary approach involving addiction services. Despite a stable postoperative course, loss to follow‑up underscores the challenges in ensuring continuity of care in this population. Early integration of addiction medicine and structured follow‑up pathways are essential to improve outcomes.

## Limitations

This report has several limitations. First, as a two‑patient case report, the findings are not generalizable. Second, the observational nature precludes establishing causality between NSAID use or polysubstance exposure and the observed pathological findings. Third, loss to follow‑up in the second patient limits assessment of long‑term outcomes. Finally, toxicology screening was not performed at presentation, which may have strengthened the association between methamphetamine use and ischemic injury.

## Conclusions

Gastric perforation at the extremes of age often reflects distinct etiologies requiring tailored approaches. In elderly patients, chronic NSAID use remains a major risk factor, and routine biopsy is essential to exclude dysplasia or malignancy. In younger patients with polysubstance use, methamphetamine‑related ischemia should be considered—even in the absence of free air on radiography—and CT imaging should be pursued early. While modified Graham patch repair remains effective, postoperative management must address the underlying condition, including oncological surveillance or addiction rehabilitation. Individualized care, multidisciplinary coordination, and careful risk–benefit assessment are key to optimizing patient outcomes.

## Data Availability

The data used and/or analyzed during the current study are available from the corresponding author upon reasonable request.

## References

[1] Søreide K, Thorsen K, Harrison EM, Bingener J, Møller MH, Ohene-Yeboah M, et al. Perforated peptic ulcer. Lancet. 2015;386(10000):1288–98. 10.1016/S0140-6736(15)00276-7. 26460663 10.1016/S0140-6736(15)00276-7 PMC4618390

[2] Lau JY, Sung J, Hill C, Henderson C, Howden CW, Metz DC. Systematic review of the epidemiology of complicated peptic ulcer disease: incidence, recurrence, risk factors and mortality. Digestion. 2011;84(2):102–13. 10.1159/000323958. 21494041 10.1159/000323958

[3] Amalia R, Vidyani A, I’tishom R, Efendi WI, Danardono E, Wibowo BP, et al. The prevalence, etiology and treatment of gastroduodenal ulcers and perforation: a systematic review. J Clin Med. 2024;13(4):1063. 10.3390/jcm13041063. 38398375 10.3390/jcm13041063 PMC10888557

[4] Wongrakpanich S, Wongrakpanich A, Melhado K, Rangaswami J. A comprehensive review of non-steroidal anti-inflammatory drug use in the elderly. Aging Dis. 2018;9(1):143–50. 10.14336/AD.2017.0306. 29392089 10.14336/AD.2017.0306 PMC5772852

[5] Jahagirdaar D, Bomanwar N, Joshi S. A prospective clinicoendoscopic follow-up study in young patients with peptic ulcer perforation at a tertiary institute in central India. Euroasian J Hepatogastroenterol. 2019;9(2):91–5. 32117697 10.5005/jp-journals-10018-1306 PMC7047314

[6] Kaul P, Koul K, Singh G, Riaz M. A clinical study of perforated peptic ulcer in young people in Jammu – a prospective study. Int J Creat Res Thoughts. 2023;11(9):e11–7.

[7] Andersen IB, Jørgensen T, Bonnevie O, Grønbaek M, Sørensen TI. Smoking and alcohol intake as risk factors for bleeding and perforated peptic ulcers: a population-based cohort study. Epidemiology. 2000;11(4):434–9. 10.1097/00001648-200007000-00012. 10874551 10.1097/00001648-200007000-00012

[8] Turan B, Eroğlu H, Sultanoğlu B, Demirbakan K. Methamphetamine-related peptic ulcer perforation: a growing medical concern. Ulus Travma Acil Cerrahi Derg. 2023;29(12):1357–63. 10.14744/tjtes.2023.53146. 38073456 10.14744/tjtes.2023.53146 PMC10767286

[9] Anderson JE, Brown IE, Olson KA, Iverson K, Cocanour CS, Galante JM. Nonocclusive mesenteric ischemia in patients with methamphetamine use. J Trauma Acute Care Surg. 2018;84(6):885–92. 10.1097/TA.0000000000001855. 29462085 10.1097/TA.0000000000001855

[10] Weledji EP. An overview of gastric perforation. J Surg Clin Pract. 2022;6(3):1–6.

[11] Takeuchi K. Pathogenesis of NSAID-induced gastric damage: importance of cyclooxygenase inhibition and gastric hypermotility. World J Gastroenterol. 2012;18(18):2147–60. 10.3748/wjg.v18.i18.2147. 22611307 10.3748/wjg.v18.i18.2147 PMC3351764

[12] Becker JC, Domschke W, Pohle T. Current approaches to prevent NSAID-induced gastropathy – COX selectivity and beyond. Br J Clin Pharmacol. 2004;58(6):587–600. 10.1111/j.1365-2125.2004.02198.x. 15563357 10.1111/j.1365-2125.2004.02198.x PMC1884640

[13] Tantinam T, Temram S, Treeratanawikran T, Kamoncharoen P, Srimaneerak E, Siripoonsap M, et al. Concurrent emphysematous gastritis and small bowel ischemia induced by methamphetamine abuse. J Health Sci Med Res. 2025;43(4):e20251144.

[14] Kumar V, Abbas AK, Aster JC. Robbins & Cotran pathologic basis of disease. 10th ed. Philadelphia (PA): Elsevier; 2020.

[15] Richards JR, Laurin EG. Methamphetamine toxicity. StatPearls [Internet]. Treasure Island (FL): StatPearls Publishing; 2026. p. 28613645. 28613645

[16] Pimentel-Nunes P, Libânio D, Marcos-Pinto R, Areia M, Leja M, Esposito G, et al. Management of epithelial precancerous conditions and lesions in the stomach (MAPS II): European Society of Gastrointestinal Endoscopy (ESGE), European Helicopter and Microbiota Study Group (EHMSG), European Society of Pathology (ESP), and Sociedade Portuguesa de Endoscopia Digestiva (SPED) guideline update 2019. Endoscopy. 2019;51(4):365–88. 10.1055/a-0859-1883. 30841008 10.1055/a-0859-1883

[17] Sung JK. Diagnosis and management of gastric dysplasia. Korean J Intern Med. 2016;31(2):201–9. 10.3904/kjim.2016.021. 26932397 10.3904/kjim.2016.021 PMC4773732

[18] Abrahami D, Pradhan R, Yin H, Yanofsky R, Gibson E, Bitton A, et al. Proton pump inhibitors and the risk of inflammatory bowel disease: population-based cohort study. Gut. 2023;72(1):3–11. 10.1136/gutjnl-2022-328866. 10.1136/gutjnl-2022-328866 36717221

[19] Leonard J, Marshall JK, Moayyedi P. Systematic review of the risk of enteric infection in patients taking acid suppression. Am J Gastroenterol. 2007;102(9):2047–56. 10.1111/j.1572-0241.2007.01275.x. 17509031 10.1111/j.1572-0241.2007.01275.x

[20] Heidelbaugh JJ. Proton pump inhibitors and risk of vitamin and mineral deficiency: evidence and clinical implications. Ther Adv Drug Saf. 2013;4(3):125–33. 10.1177/2042098613482484. 25083257 10.1177/2042098613482484 PMC4110863

